# Mechanical Behavior and Failure Modes of Cemented Backfill Under Impact Loading

**DOI:** 10.3390/ma19112219

**Published:** 2026-05-25

**Authors:** Xiaohua Zhang, Zhiyong Yang, Xianglong Li, Fuming Liu, Defeng Hou, Ting Zuo, Jianguo Wang

**Affiliations:** 1 Faculty of Land Resources Engineering, Kunming University of Science and Technology, Kunming 650093, China; sunset830@163.com (X.Z.); lxl00014002@163.com (X.L.); 2 Xinjiang Tianchi Energy Co., Ltd., Changji 831700, China; yzy912@163.com (Z.Y.); fumingliuok@126.com (F.L.); 18811752600@163.com (D.H.); 3 Xinjiang Green Blasting Engineering Technology Research Center, Changji 831700, China; 4 Advanced Blasting Technology Engineering Research Center of Yunnan Province Education Department, Kunming 650093, China

**Keywords:** cemented backfill, dynamic response, microstructure, crack propagation, fractal dimension

## Abstract

Cemented backfill (CTB) in mining operations is frequently exposed to blasting-induced dynamic disturbances. These disturbances compromise CTB stability. This study employed split Hopkinson pressure bar (SHPB) tests, scanning electron microscopy (SEM), and high-speed imaging to characterize dynamic mechanical response and failure modes of CTB. The results showed that dynamic compressive strength (DCS) increased with impact load. However, damage severity also increased with load. High-speed imaging of crack evolution yielded fractal dimensions between 0.5 and 1.3. Higher fractal dimensions correlated with more extensive macroscopic cracking and greater CTB damage. They also indicated an increased probability of CTB failure and loss of load-bearing capacity. Microstructural observations identified ettringite (AFt) and C-S-H gel as the principal hydration products that provided cohesion and strength. However, pervasive microcracks and micropores accounted for the CTB’s low inherent strength. The findings suggest that reducing dynamic loads during mining operations can mitigate damage to CTB.

## 1. Introduction

**Figure 1:**
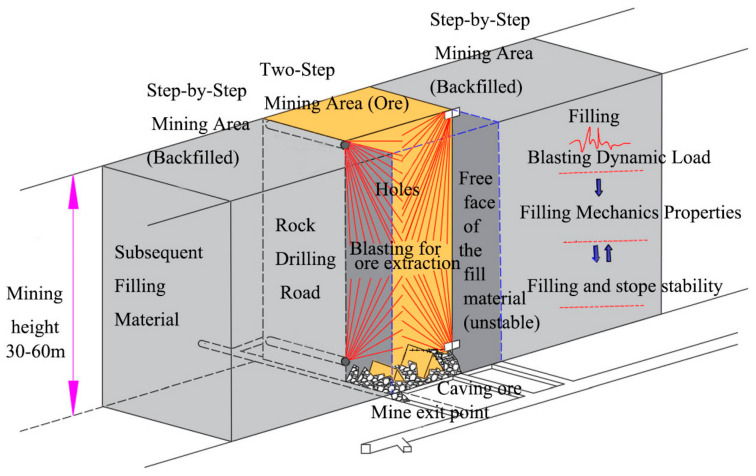
Schematic diagram of stress of backfill in two-step mining.

Mining operations create numerous goafs that can cause surface subsidence, stope instability, and other geological hazards. Post-extraction backfilling has therefore been widely adopted to mitigate these adverse impacts [1,2]. This method typically uses relatively large stage heights. As a result, the margins of cemented backfill (CTB) are exposed to blasting-induced dynamic loads from adjacent stope extraction during the secondary mining stage. These dynamic loads directly compromise the stability of neighboring backfill and consequently degrade stope stability and mining productivity [3,4] (Figure 1). Clarifying the dynamic mechanical behavior and fracture mechanisms of CTB under dynamic loading is essential for designing effective backfill protection measures during two-stage extraction and for ensuring mine safety and resource recovery.

The split Hopkinson pressure bar (SHPB) is the primary experimental method for studying the dynamic behavior of CTB. Ma et al. [5] performed dynamic tests on single- and double-layer backfill. They found that double-layer configurations exhibited superior stability, and the rate of strain accumulation decreased with increasing strain rate. Tan et al. [6] reported that repeated cyclic impacts caused densification and sealing of pores and cracks, which increased dynamic compressive strength (DCS) and load-bearing capacity. Zhu et al. [7] proposed a dynamic strength enhancement coefficient that correlates strongly with strain rate for backfill. Cao [8,9] investigated dynamic mechanical behavior and failure propagation mechanisms of CTB using SHPB tests and LS-DYNA simulations. They found that dynamic peak stress and specimen fragmentation both increased with mean strain rate. Chen et al. [10] found that increasing cement, tailings content, and curing age enhances the dynamic strength of backfill, with dynamic strength exhibiting an exponential correlation with strain rate. Zou et al. [11] studied the dynamic mechanical properties of polypropylene fiber-reinforced CTB under triaxial impact loading, showing that strain rate and confining pressure significantly enhance strength and alter failure modes. Tan et al. [12] observed that backfill can exhibit multiple stress peaks under impact loading, with dynamic strength increasing with higher cement content and strain rate. Li et al. [13,14] analyzed the mechanical properties and microstructure of CTB and identified changes in pore structure and sulfate adsorption on C-S-H as key factors controlling backfill strength. These studies offer valuable references for understanding the dynamic behavior of CTB.

Numerous studies have investigated how backfill strength evolves under dynamic loading. Song et al. [15] performed SHPB impact tests on CTB to characterize its dynamic mechanical response to blasting perturbations. They examined the evolution of CTB’s compressive strength and damage characteristics as a function of curing age and rice straw content. Hou et al. [16] performed SHPB impact tests on CTB under varying strain rates and found that DCS increased exponentially with strain rate. Xu et al. [17] studied how cement content and interface angle affect the dynamic behavior of stratified tailings backfill. Xue et al. [18] employed SHPB and high-speed imaging to assess the impact of fiber content on the DCS of backfill. Ma et al. [19] found that confining pressure and strain rate exhibit a mutually inhibitory coupling effect on rock strength and established an accurate prediction criterion. Krzaczek and Nitka et al. [20,21] pointed out, through DEM/CFD and breakage modeling, that strain rate, aggregate fragmentation, and free water all enhance the dynamic strength of concrete, with free water retarding the fracture process via pore pressure. Li et al. [22] and Robinson et al. [23] demonstrated for clays that the maturity of cementation structure, drainage mechanism, and liquidity index significantly influence the sign and magnitude of the strain rate effect, which decreases with increasing strain level.

Kammer et al. [24] pointed out the cross-scale challenges in defining and measuring earthquake energy dissipation within a fracture mechanics framework, emphasizing the need to distinguish dissipation near the rupture tip from that far behind it. Li et al. [25] and Wang et al. [26] studied sandstone and tuff, respectively, and found that water content alters the strength, failure mode, and energy dissipation of rocks. Saturated specimens exhibit reduced energy storage and absorption capacity, but the “Stefan” effect at high strain rates leads to a greater increase in dissipation energy density, accompanied by a higher fractal dimension of crack growth. Zou et al. [27] investigated fiber-reinforced cemented tailings backfill and showed that confining pressure weakens the strain rate strengthening effect. Dissipated energy density increases with confining pressure, while the energy dissipation rate decreases with increasing strain rate, and the fractal dimension exhibits complex functional relationships with various parameters.

**Figure 2:**
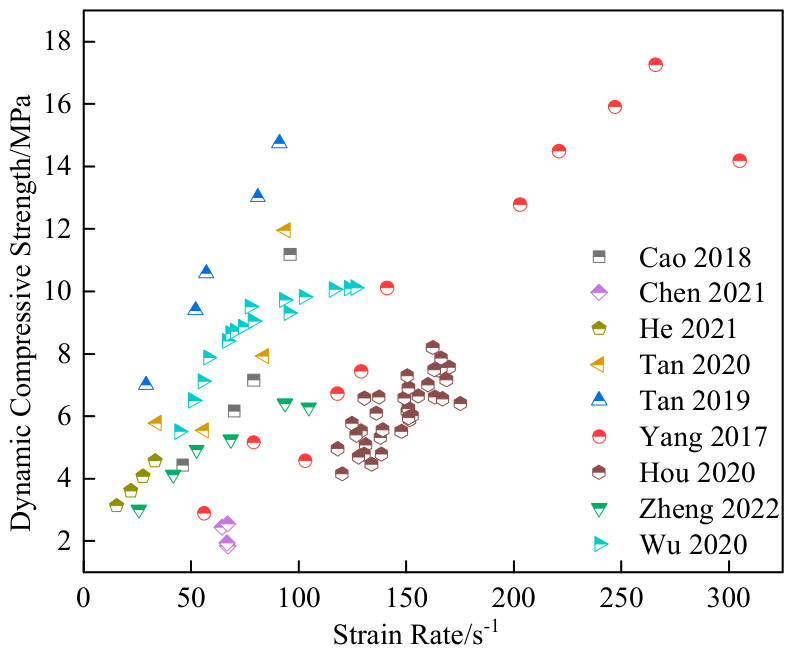
Relationship between DCS of CTB and strain rate [10,12,27,28,29,30,31,32,33].

Figure 2 summarizes the reported DCS measurements for CTB with a cement-to-sand ratio of 1:4 and a 75% solid mass concentration under blasting-related loading conditions [28,29,30,31,32,33,34]. The data show a clear dependence of DCS on strain rate. DCS increases substantially with increasing impact on the strain rate and then tends to plateau beyond a threshold strain rate.

Previous research has focused mainly on the effect of curing age on the dynamic mechanical properties of CTB. Investigations into macroscopic crack-failure mechanisms and the underlying micromechanical processes remain limited. To address this knowledge gap, this study employed SHPB, SEM, and high-speed imaging to characterize the dynamic response and failure modes of CTB. Dynamic mechanical properties were measured under different dynamic loads, and the temporal evolution of macroscopic crack propagation was analyzed. The results provide guidance for blasting designs aimed at preserving CTB thickness.

## 2. Experimental Setup

### 2.1. Experimental Apparatus

**Figure 3:**
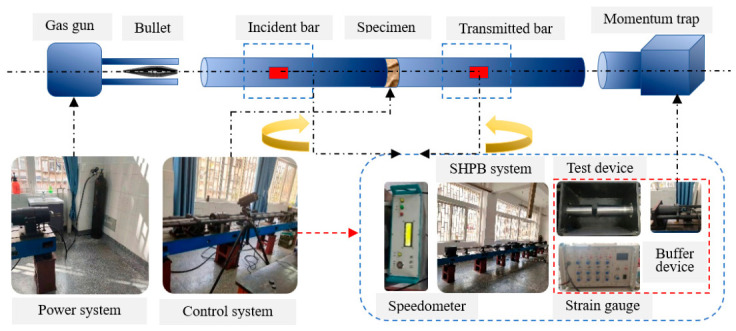
SHPB test system.

Tests were conducted on the SHPB apparatus at Kunming University of Science and Technology (Figure 3). The apparatus consists of a spindle-shaped striker, an incident bar, a transmitted bar, a loading system, and a data acquisition system. The incident and transmission bars are alloy steel with an elastic modulus of 210 GPa, a longitudinal wave speed of 5190 m/s, and a density of 7.85 g/cm^3^. The striker length is 0.35 m, and both the incident and transmission bars are 2.0 m long.

### 2.2. Specimen Preparation and Test Conditions

**Figure 4:**
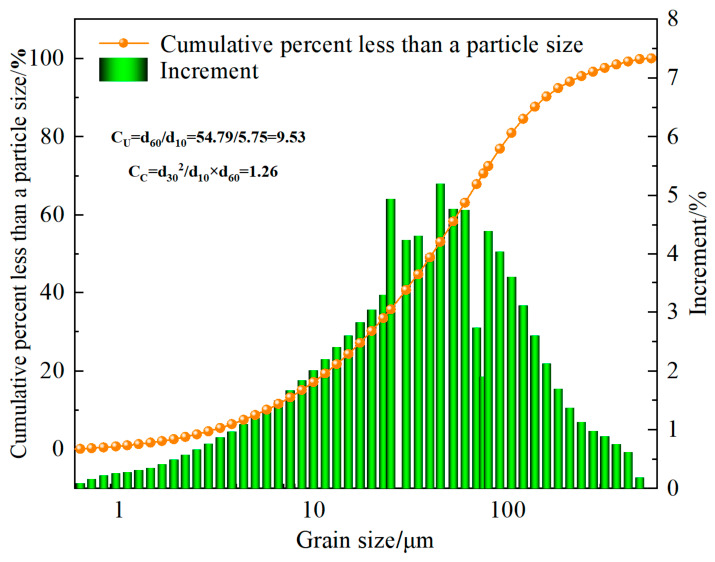
Particle size distribution of tailings.

Tailings for specimen preparation were collected from a copper mine in Yunnan Province. Physical and chemical properties were measured using a Malvern laser particle-size analyzer and X-ray fluorescence (XRF) spectroscopy. Figure 4 presents the particle size distribution. Table 1 lists the chemical composition. Particles smaller than 74 μm and 20 μm account for 70.13% and 30.15% of the sample, respectively. The mean particle diameter *d* is 40.93 μm. Ordinary Portland cement and municipal tap water were used for mixing. *C*u denotes the coefficient of uniformity. Higher *C*u values indicate a more uneven particle size distribution. *C*c denotes the coefficient of curvature and reflects the continuity of the particle size distribution. Materials with discontinuous gradation are more susceptible to segregation. Generally, a *C*u in the range of 5-10 and a *C*c in the range of 1-3 indicate well-graded material. Figure 4 presents *C*u = 9.53 and *C*c = 1.26. These values indicated that the copper-mine tailings exhibited a broad particle-size distribution with pronounced interdependence among size fractions.

**Figure 5:**
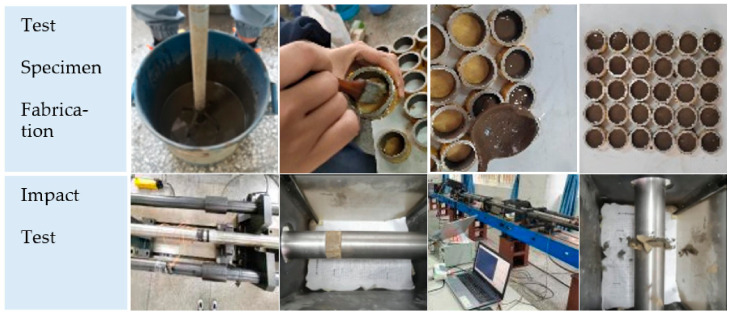
Specimen preparation and experimental procedure.

A cement-to-tailings mass ratio of 1:4 and a solid mass concentration of 75% were selected based on the mine’s current fill practice and previous experimental data [35]. Tailings and cement were thoroughly mixed, after which water was added to achieve the prescribed mix design. The mixture was then stirred in a mechanical mixer for six minutes to ensure homogenization. The homogenized paste was cast into acrylic molds (50 mm × 25 mm). Specimens were demolded after 24 h and cured in a humidity chamber at 20 °C and 90% relative humidity for 28 days. Specimens that satisfied the required strength criteria were chosen for uniaxial impact testing. The full experimental procedure is presented in Figure 5.

### 2.3. Test Methods

#### 2.3.1. Impact Test

The study examined how varying impact loads affect the dynamic mechanical behavior and failure modes of the CTB. Before testing, specimen end faces were ground using sandpaper to ensure sufficient flatness and minimize measurement errors. Cylindrical specimens measured 50 mm in diameter and 25 mm in height. A thin layer of grease was applied to specimen ends prior to testing to ensure intimate contact with the loading rods and to reduce friction-induced errors.

A nitrogen-gas-driven striker impacts the incident bar and generates an incident stress wave that propagates along the bar to the specimen-bar interface. Part of the wave transmits into the transmission bar and is attenuated by the buffer device, while the remainder is reflected back into the incident bar. Recorded data were processed after testing. Relevant parameters were computed using Equations (1)–(3) using one-dimensional stress-wave theory and the assumption of stress uniformity [36].

**Figure 6:**
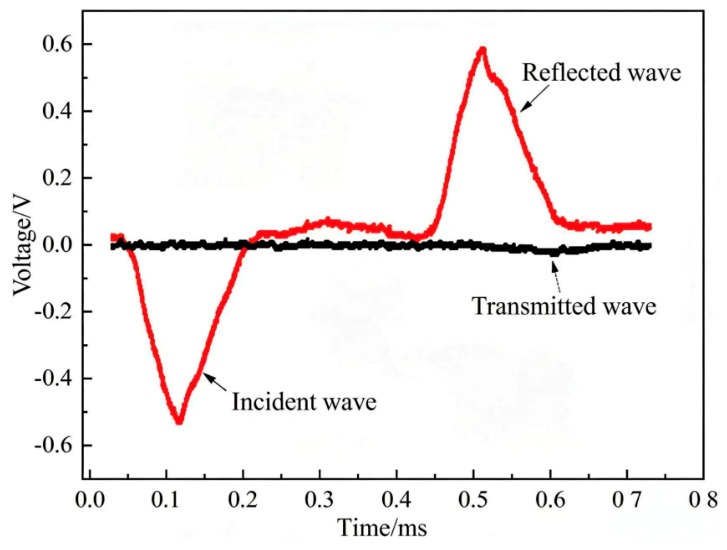
Original waveforms.

**Figure 7:**
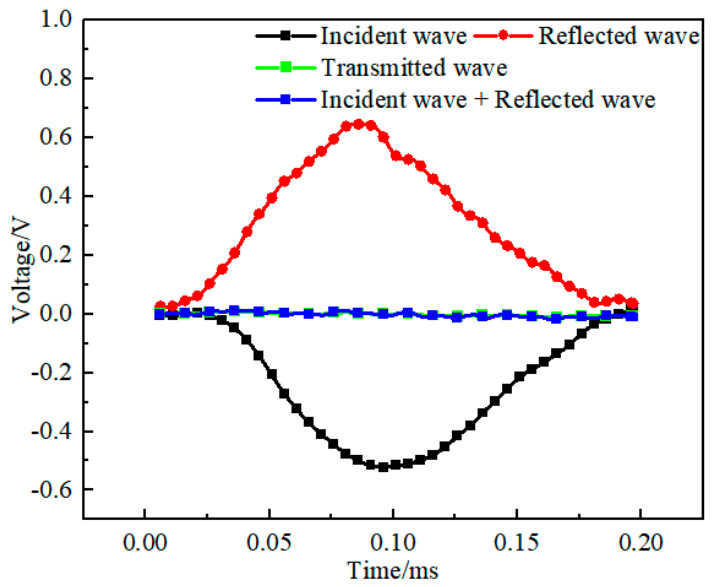
Dynamic stress equilibrium of the backfill specimen.

Figure 6 presents original waveforms for representative CTB specimens. The incident and reflected waves had nearly identical amplitudes and opposite polarities. Both amplitudes were substantially larger than that of the transmitted wave. This behavior arose because the wave impedance of the CTB was much lower than that of the incident rod. Consequently, when the incident wave reached the CTB, its energy was rapidly dissipated. Figure 7 shows the specimen data that satisfied the stress equilibrium condition. The stress curve at the incident end of the specimen (incident wave + reflected wave) is basically coincident with the stress curve at the transmitted end, indicating that uniform dynamic stress equilibrium is achieved inside the specimen during loading, and the test data are valid and available.

(1) $${\overset{•}{\varepsilon }}_{s}=\frac{C}{L_{s}}\left[\varepsilon_{i}(t)-\varepsilon_{r}(t)-\varepsilon_{t}(t)\right]$$

(2) $$\varepsilon =\frac{C}{L_{s}}\int_{0}^{t_{0}}\left[\varepsilon_{i}(t)-\varepsilon_{r}(t)-\varepsilon_{t}(t)\right]dt$$

(3) $$\sigma_{s}=\frac{EA}{2A_{s}}\left[\varepsilon_{i}(t)+\varepsilon_{r}(t)+\varepsilon_{t}(t)\right]\;$$

where *E* is the elastic modulus of the elastic compression bar; *L*_s_ is the specimen height; *A* is the diameter of the incident bar; *As* is the specimen cross-sectional area; *C* is the longitudinal wave speed; $\varepsilon_{i}$ is the incident strain; $\varepsilon_{r}$ is the reflected strain; $\varepsilon_{t}$ is the transmitted strain; ${\overset{•}{\varepsilon }}_{s}$ is the specimen strain rate; ε is the specimen strain; $\sigma_{s}$ is the specimen stress.

#### 2.3.2. SEM Analysis

**Figure 8:**
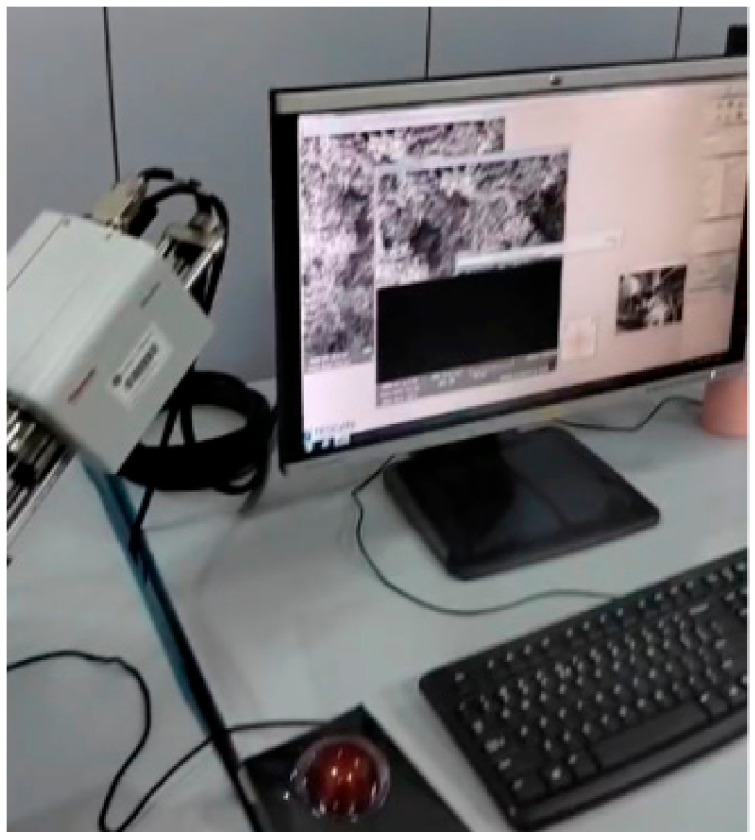
Scanning electron microscopy.

Figure 8 presents SEM images of the CTB microstructure. Due to the CTB’s low electrical conductivity, specimens were gold-coated prior to scanning, and microstructural observations were performed under vacuum conditions.

## 3. Results and Discussion

The impact velocity range was determined through preliminary impact tests. The projectile exit velocity was controlled by adjusting its initial position. The minimum impact velocity was defined as the lowest exit velocity that produced recordable data, and the maximum impact velocity was defined as the velocity that caused complete fragmentation of the cemented tailings backfill (CTB) specimen. Preliminary test results indicated an impact velocity range of approximately 3-5 m/s, with the minimum velocity being 3 m/s and the maximum velocity 5 m/s. A total of 21 test groups were completed within this range. Three parallel tests were performed for each impact velocity. The final selected results are presented in Table 2.

### 3.1. Dynamic Mechanical Behavior of the CTB Specimens

#### 3.1.1. Strength of the CTB Specimens

**Figure 9:**
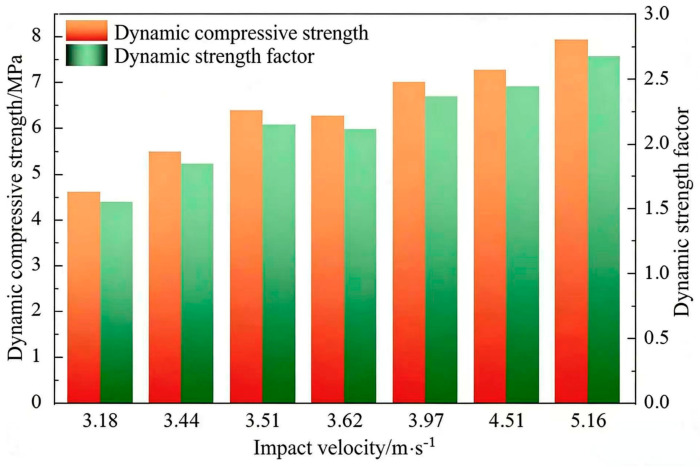
Relationship between DCS, DIF and impact velocity.

Figure 9 depicts how DCS and the dynamic increase factor (DIF) of the CTB specimens vary with impact velocity. DIF characterizes material impact resistance and quantifies strength changes under dynamic loading. DCS increased from 4.61 MPa at 3.18 m/s to 7.92 MPa at 5.16 m/s. This change corresponded to a 71.8% increase across the tested velocity range. DCS therefore exhibited a strong dependence on impact velocity. Thus, DIF was used to quantify the increase in DCS relative to the static compressive strength, as defined in Equation (4).

(4) $$K=\sigma_{d}/\sigma_{c}$$

where *K* is the dynamic increase factor; $\sigma_{d}$ is the dynamic compressive strength (MPa); $\sigma_{c}$ is the static compressive strength (MPa). Here, $\sigma_{c}$ is taken as 2.97 MPa.

The calculated K ranged from 1.55 to 2.67 for impact velocities of 3.18-5.16 m/s. This behavior is attributed to the CTB’s low strength, poor compactness, and abundant inherent fissures. Increasing impact velocity (strain rate) raised the incident energy and the energy absorbed by the CTB. However, the short duration of the impact load reduced the time available for energy dissipation. According to the work-energy principle, the CTB must withstand external impact forces by increasing its internal stresses. Consequently, its DCS increased markedly [17].

From the perspective of microcrack propagation: Under low impact loading, cracks in the backfill propagate preferentially along weak structures such as pores and interfaces to form penetrating main cracks, and the strength is dominated by static fracture toughness. With the increase in loading rate, cracks fail to develop along optimal paths, while a large number of internal microcracks initiate and bifurcate simultaneously and restrict one another, which effectively improves the macroscopic strength. When the strain rate further increases, the microcrack density reaches saturation and rapidly coalesces, the specimen suffers crushing failure, the strength approaches the ultimate bearing capacity of the material, and its growth rate gradually slows down.

From the perspective of inertial confinement effects: During SHPB loading at high strain rates, the backfill produces an obvious radial inertial constraint effect and forms an equivalent passive confining pressure, which effectively restrains the lateral expansion deformation of the specimen. Meanwhile, the inertial effect hinders the rapid penetration and expansion of macroscopic cracks, collectively leading to a remarkable increase in the dynamic compressive strength of backfill with the rising strain rate.

#### 3.1.2. Stress-Strain Curves of the CTB Specimens

**Figure 10:**
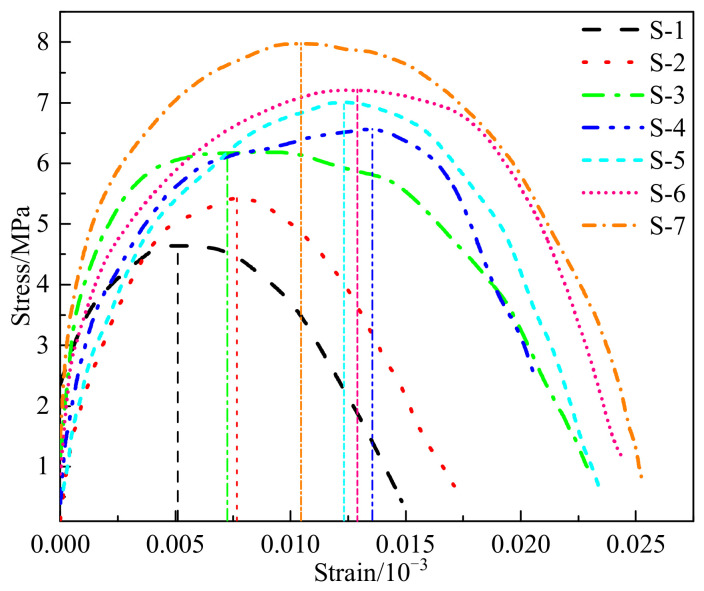
Stress-strain curve.

Figure 10 presents stress-strain curves for CTB specimens under varying impact loads.

The specimen’s stress-strain curves exhibited nearly identical initial ascending segments. This indicated comparable elastic moduli during the early stage. As the impact velocity increased, the slope of the ascending segments increased gradually. This trend indicated a modest rise in elastic modulus.

All specimens exhibited a consistent sequence of deformation stages that can be comparatively characterized from the stress-strain response. Initially, internal microcracks closed, corresponding to a compaction stage with a limited strain range, which was much less pronounced than that under static loading. The specimen then entered the elastic stage, indicated by the upward convex stress-strain curve and a slight increase in slope with impact velocity, with no evident crack propagation.

Subsequently, the specimen transitioned into the plastic-yielding stage, marked by deviation from linearity and progressive crack propagation. Stress and damage gradually localized until peak stress was reached. Finally, a stress-unloading stage occurred, characterized by rapid stress drop and continued crack propagation, leading to unstable failure.

In terms of failure modes, the observed behavior is consistent with typical dynamic failure models of brittle geomaterials, showing a transition from tensile splitting at low strain rates to crushing-dominated failure at high strain rates.

#### 3.1.3. Impact-Induced Deformation and Failure Behavior of the CTB

**Figure 11:**
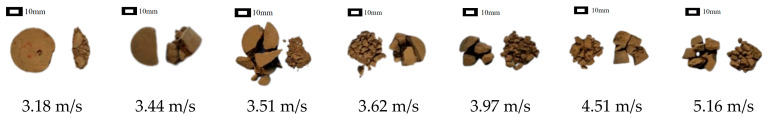
Fragmentation morphology of CTB specimens under different impact velocities.

CTB is a multiphase composite containing macroscopic defects, such as cracks and voids, which define its weakest regions. The CTB specimen experienced minor damage at an impact velocity of 3.18 m/s, including localized microcracks and small-scale fragmentation. When the impact velocity exceeded 3.62 m/s, the CTB specimen developed tensile cracks and transitioned toward a tensile-shear failure mode. Higher mean strain rate produced more extensive macroscopic cracks and deeper fragmentation. This study further elucidated the fragmentation morphology of CTB specimens across different strain rates. Figure 11 presents the macroscopic fragmentation morphologies observed at various impact velocities.

Damage to the CTB intensified as impact velocity increased. The proportion of large fragments decreased, while fine fragments became more abundant. These observations indicated a pronounced strain-rate effect. Failure mode shifted from edge spalling and axial tensile splitting at low velocities to crush-type fracturing with smaller fragment sizes at high velocities. This trend agrees with the results of Sun et al. [37]. This phenomenon occurred because most of the energy absorbed by the CTB was expended on nucleation, growth and propagation of internal microcracks under low impact loads. The macroscopic manifestation of this process was edge spalling and axial tensile splitting. When the impact load was relatively high, the energy absorbed by the CTB was consumed in generating more new microcracks, which intersected and coalesced with the initial microcracks. Consequently, this process intensified damage to the CTB.

### 3.2. Energy Variation in the CTB

The deformation and failure of the CTB under external impact loading represent the processes of energy conversion and transfer. The expressions for the specimen’s incident energy $W_{I}$, reflected energy $W_{R}$, transmitted energy $W_{T}$, and absorbed energy $W_{S}$ are given by:

(5) $$\left\{\begin{array}{c} W_{I}(t)=AEC\int_{0}^{t}\varepsilon_{i}^{2}(t)dt\\ W_{R}(t)=AEC\int_{0}^{t}\varepsilon_{r}^{2}(t)dt\\ W_{T}(t)=AEC\int_{0}^{t}\varepsilon_{t}^{2}(t)dt \end{array}\right.$$

(6) $$W_{s}=W_{I}-\left(W_{R}+W_{T}\right)$$

Based on the rock-mechanics definition of absorbed energy per unit volume, the specific energy absorption, $\omega_{cp}$, was introduced to quantify the absorbed energy per unit volume of the CTB. It is defined as follows [38]:

(7) $$\omega_{cp}=\frac{W_{s}}{V_{s}}$$

where $V_{s}$ is the specimen volume (cm^3^).

Additionally, the specific energy consumption per unit mass, $\omega_{d}$, was introduced to quantify the energy dissipation by the CTB. It is given by:

(8) $$\omega_{d}=\frac{W_{s}}{M_{s}}$$

where $M_{s}$ is the specimen mass (g).

**Figure 12:**
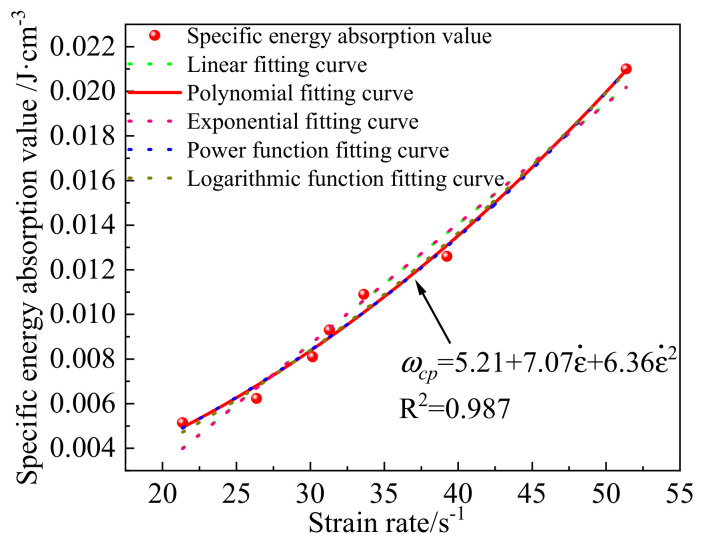
Relationship between specific energy absorption of CTB and strain rate.

**Figure 13:**
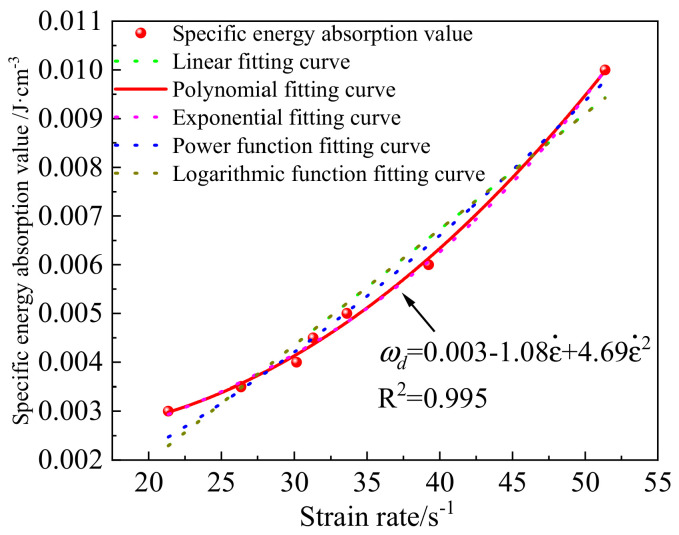
Relationship between crushing energy consumption per unit mass of CTB and strain rate.

The specific energy absorption and the unit-mass crushing energy of the CTB under dynamic loading were calculated using Equations (7) and (8). The results are presented in Figure 12 and Figure 13. Both variables exhibited a clear dependence on strain rate. Fits using linear, exponential, power, and polynomial functions revealed a strong quadratic relationship between the two variables (R^2^ > 0.98). Specific energy absorption and unit-mass crushing energy increased with increasing impact velocity.

Under dynamic impact, the energy evolution of cemented backfill is primarily governed by the initiation, propagation, and macroscopic evolution of internal cracks. At low strain rates, pre-existing defects and microcracks propagate slowly, with some cracks even partially closing. The material mainly experiences minor damage, characterized by a limited fracture range and low energy dissipation. As a result, both the specific energy absorption and the unit mass crushing energy increase gradually. As the strain rate increases, the dynamic loading effect becomes more pronounced. Existing cracks rapidly propagate and interconnect, while numerous secondary microcracks are continuously generated. The fracture mode transitions from single-crack propagation to the coordinated development of multiple cracks, forming a complex crack network. Under high strain rates, crack propagation accelerates further, and fragmentation becomes more severe. The formation of numerous new cracks and multi-level fracture surfaces consumes substantial energy, leading to a rapid increase in both specific energy absorption and unit mass crushing energy.

## 4. Macro- and Micro-Scale Surface Features of the CTB

### 4.1. Evolutionary Patterns of Crack Propagation in CTB

#### 4.1.1. Crack Propagation Characteristics

**Figure 14:**
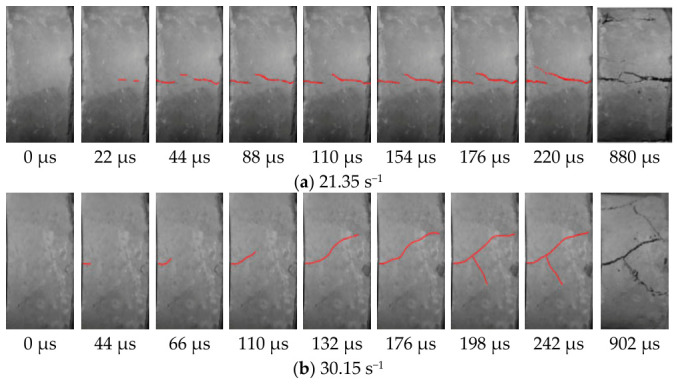

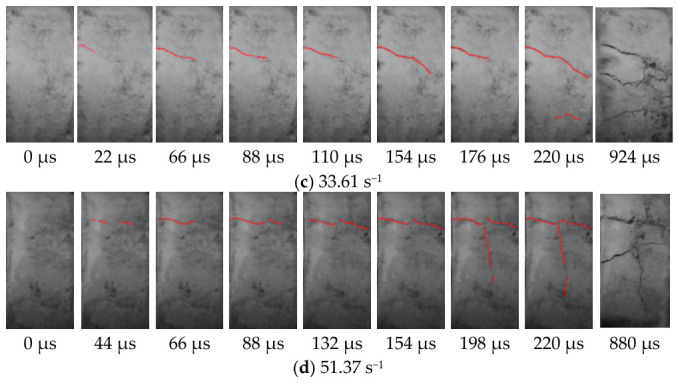
Crack propagation process in CTB.

Dynamic SHPB impact tests on the CTB specimens were recorded using a MotionPro Y7 high-speed camera with supplementary lighting, manufactured by Integrated Design Tools, Inc., Pasadena, CA, USA. Figure 14 presents sequences of crack development during dynamic compression at four increasing strain-rate levels. These photos illustrate crack initiation, propagation, and evolution under varying impact-gas pressures.

Figure 14 shows that specimen stress increased continuously during stress-wave loading. Initial cracks formed immediately after the peak compressive strength was reached and propagated parallel to the loading axis (marked by red lines). As the loading proceeded, the cracks extended axially and developed into a primary crack. Before the primary crack fully penetrated the specimen, the contact region between the incident end and the specimen experienced combined compressive and shear stresses. Consequently, the primary crack branched and generated secondary cracks and a distinct fracture plane, which led to complete crushing of the specimen. The specimen exhibited typical splitting failure characteristics.

#### 4.1.2. Fractal Features of Impact-Induced Cracks in CTB

Numerous studies [39,40,41] indicate that crack fractal characteristics provide a quantitative measure of specimen damage. The macroscopic unstable failure of CTB in both field mining and laboratory experiments can be regarded as an energy dissipation process. The failure of the CTB progresses through the propagation of numerous small cracks, each of which originates from finer-scale cracks. This hierarchical pattern conforms to the self-similarity principle of fractal theory. Consequently, microcracks formed during CTB failure exhibited pronounced fractal characteristics. This study applied fractal-dimension analysis to characterize CTB failure under dynamic compression in SHPB tests. The temporal evolution of the apparent fractal dimension during dynamic fragmentation was also investigated.

Fractal dimensions of impact-induced surface cracks at successive failure stages were calculated using the box-counting method implemented in MATLAB R2025b, which was also used for all data processing and numerical simulations in this study. To ensure the accuracy of fractal dimension calculations, this study applied a unified preprocessing procedure to the surface crack images of CTB specimens after impact. First, grayscale conversion, median filtering for noise reduction, and histogram equalization for contrast enhancement were performed. Subsequently, the Otsu method was adopted to automatically select a global threshold for image binarization, thereby separating the crack regions from the background. Crack extraction was carried out using a combination of manual and automatic approaches: after automatic segmentation, the binary images were inspected, and pseudo-cracks caused by surface stains or uneven illumination were manually removed to ensure the integrity of the crack structure. All image processing and fractal dimension calculations were implemented using self-written MATLAB programs. It should be noted that the calculated fractal dimension depends on image resolution; therefore, all images in this study were uniformly sampled at 960 × 1920 pixels, and the grid sizes δ were taken as integer powers of 2 to ensure comparability of the results.

These fractal dimensions were then used to quantitatively assess the extent of specimen damage. The crack map was covered with squares of side length *δ*. Some boxes contained no crack, while the others were fully occupied by the cracks. Let *N*(*δ*) denote the number of nonempty boxes. The box-counting fractal dimension *D* is defined as the limit *δ* → 0:

(9) $$D=-\underset{\delta \to 0}{lim}\;\frac{lg\;N(\delta )}{\mathrm{lg}\delta }$$

Because *δ* was selected from a finite set in the computations, the corresponding pairs (*δ*, *N*(*δ*)) were obtained. Fitting the log-transformed data in a log-log plot yielded Equation (10). The slope *D* of the resulting linear fit corresponds to the fractal dimension [42].

(10) lgN(δ)=Dlgδ+b

**Figure 15:**
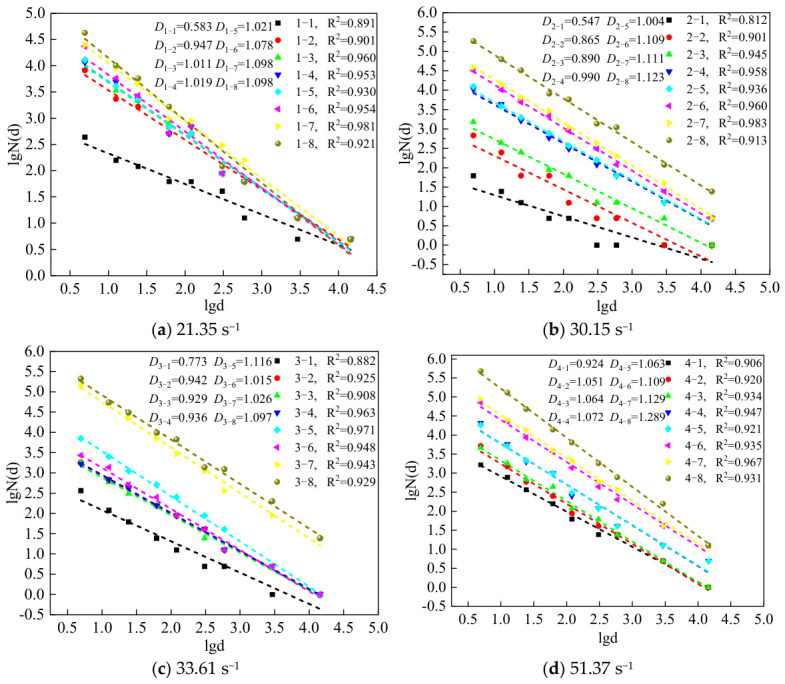
Linear fits of the box-counting fractal dimension of impact-induced cracks.

Binary images of impact-induced cracks were partitioned into grids and analyzed in MATLAB. The box-counting method was applied to compute the fractal dimension of each computational region. Figure 15 presents the linear-fit plots of the box-counting fractal dimension of impact-induced cracks in CTB specimens at different strain rates and time points.

Label 1-1 in Figure 15a corresponds to the second high-speed photograph describing crack propagation in the first group (see the previous section). The first photograph captured at the moment of impact showed no crack propagation and was therefore excluded from the calculations.

Fitting results indicated that the logarithm of the number of the square boxes was linearly related to the logarithm of the number of the boxes corresponding to the measured object. This finding indicated pronounced fractal characteristics in the evolution of surface cracks in CTB specimens under impact loading. In addition, the slope of the log–log plot increased with impact velocity. This result indicated a corresponding increase in fractal dimension.

**Figure 16:**
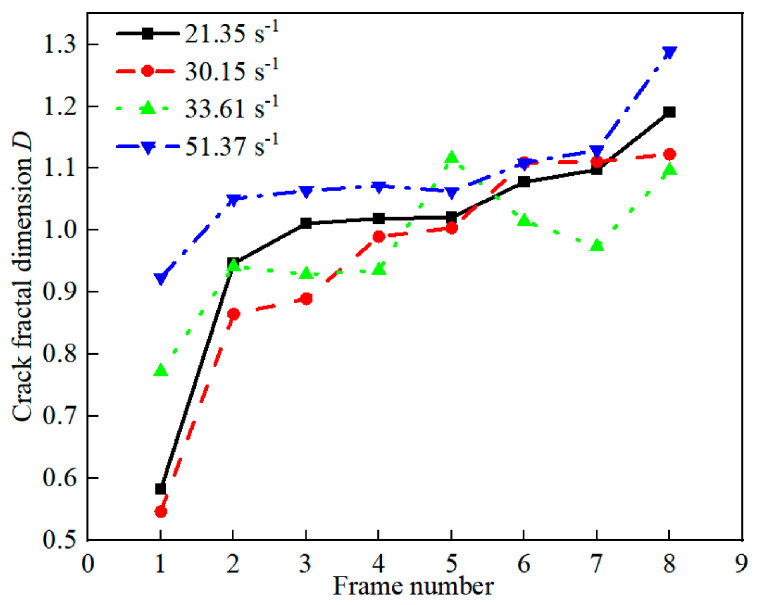
Variation in fractal dimension of cracks in CTB with impact velocity.

Figure 16 demonstrated a continuous increase in the fractal dimension of surface cracks in CTB specimens under impact loading. Fractal dimension values ranged from 0.5 to 1.3. This result indicated a high degree of crack dispersion during impact-induced failure. As the impact load increased, the apparent fractal dimension also increased. This observation indicated more complex crack propagation paths and more severe damage.

At a low strain rate, the fractal dimension of the CTB specimen is relatively low, corresponding to the slow development of a small number of internal microcracks, which maintains favorable structural integrity and induces slight degradation of mechanical properties. As the impact velocity increases, the fractal dimension rises significantly, which intuitively reflects the whole evolutionary process of extensive initiation of secondary cracks, cross-penetration of crack systems, and gradual complication of fracture surfaces inside the specimen.

The dynamic increase in fractal dimension essentially quantifies the progressive internal damage accumulation, attenuation of structural integrity, and continuous degradation of bearing capacity, which can be adopted as a direct indicator to characterize the dynamic damage degree of cemented tailings backfill (CTB). A higher fractal dimension corresponds to more developed internal fissures and defects, a substantially reduced effective bearing area, and deteriorated structural stability. Accordingly, the possibility of overall instability and fragmentation of CTB specimens under dynamic loading is greatly increased.

### 4.2. Microstructure Analysis of the CTB

Scanning electron microscopy (SEM) was employed to analyze the post-impact microstructure of CTB specimens to elucidate mechanisms responsible for the degradation of mechanical properties. SEM provides multi-angle imaging of microstructural features and enables detailed characterization of fracture morphologies associated with CTB failure.

**Figure 17:**
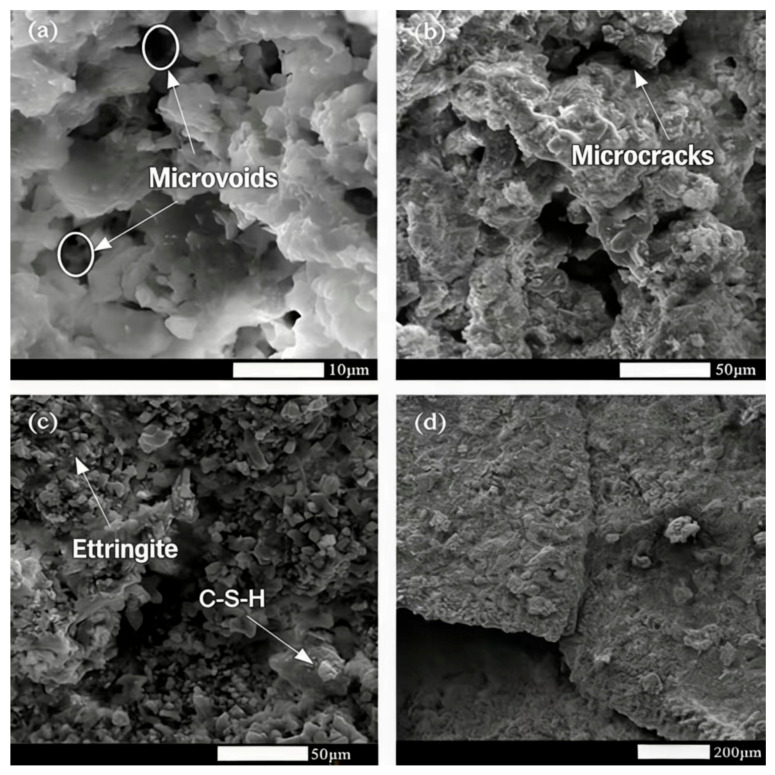
Microstructure of cemented tailings backfill (CTB): (**a**) Micropore morphology, (**b**) Microcrack characteristics, (**c**) Hydration product microstructure, (**d**) Macroscopic fracture morphology.

As shown in Figure 17, the cemented tailings backfill (CTB) contains abundant flocculent calcium silicate hydrate (C-S-H) gel and needle-like ettringite (AFt), which constitute the primary cementitious skeleton of CTB. The continuous matrix formed by C-S-H gel, together with AFt crystals filling the pore gaps, effectively bonds tailings particles and improves the matrix compactness and bearing capacity.

Under low impact loading, the inherent micropores and microcracks inside CTB are compacted and closed, resulting in slight matrix densification. As the impact velocity increases, the cementitious interfaces fail to withstand dynamic loading. Secondary cracks rapidly initiate, propagate and coalesce along pre-existing defects, leading to the fracture of the integrated C-S-H/AFt hydration skeleton. This significantly weakens the cementation effect and severely impairs the structural integrity of CTB.

The progressive microstructural deterioration is highly consistent with the macroscopic quadratic increase in energy dissipation and the remarkable growth in fractal dimension. The continuous development of fracture networks consumes substantial impact kinetic energy, thereby sharply increasing the specific energy absorption and unit-mass crushing energy of CTB.

## 5. Conclusions

This study employed SHPB to perform uniaxial impact compression tests on CTB specimens. High-speed imaging and SEM were employed to capture dynamic failure behavior and characterize microstructural features. The effects of different impact loads on DCS, stress-strain response, failure modes, energy evolution, and structural damage patterns at both macro and microscales were investigated. The main conclusions are as follows.

(1) Within the impact velocity range of 3–5 m/s, the dynamic compressive strength (DCS) of CTB increased from 4.61 MPa to 7.92 MPa, while the dynamic increase factor (DIF) rose from 1.55 to 2.67, demonstrating a pronounced strain-rate effect. Meanwhile, both the specific energy absorption and the unit-mass crushing energy exhibited a quadratic relationship with strain rate, indicating a nonlinear enhancement in energy dissipation capacity.
(2) As the mean strain rate increased, crack initiation occurred earlier, and both the number and propagation rate of cracks increased significantly. The failure mode gradually transitioned from axial tensile splitting at low velocities to crushing failure at high velocities. The fractal dimension of cracks increased from 0.5 to 1.3, reflecting the increasing complexity of the crack network and the intensification of damage, which corresponded to the degradation of the load-bearing capacity.
(3) Microstructural analysis revealed that the primary hydration products of CTB are C-S-H gel and AFt, which together form the cementitious skeleton and provide strength. However, the widespread presence of micropores and microcracks is the fundamental cause of its low inherent strength. Under dynamic loading, cracks initiate, propagate, and coalesce along pre-existing defects, leading to the destruction of the cementitious skeleton and a significant reduction in structural integrity.
(4) Under blasting-induced dynamic loading, the damage evolution of CTB is governed by the strain rate. Therefore, the effects of impact intensity (e.g., explosive charge and distance from the blast source) on dynamic strength, crack propagation, and energy dissipation characteristics should be comprehensively considered to reduce the risk of instability in the backfill.

## Figures and Tables

**Table 1:** Chemical constituents of tailings.

Component	CaO	MgO	SiO_2_	Al_2_O_3_	S	Cu	Zn	TFe
Content/%	5.96	2.08	45.89	12.32	0.5	0.035	0.067	11.06

**Table 2:** Test results.

Number	*v*/ (m·s^−1^)	Specimen Dimensions		$\sigma_{d}$/MPa	Dynamic Increase Factor (DIF)	Failure Modes
		***D*/mm**	***H*/mm**			
S-1	3.18	49.36	25.12	4.61	1.55	Axial splitting failure
S-2	3.44	49.29	25.13	5.48	1.84	Axial tensile failure
S-3	3.51	49.23	25.19	6.38	2.14	Axial tensile failure
S-4	3.62	49.32	25.02	6.27	2.11	Tensile failure
S-5	3.97	48.98	24.69	7.01	2.36	Tensile failure
S-6	4.51	49.36	24.98	7.26	2.44	Tensile failure
S-7	5.16	49.67	24.88	7.92	2.67	Crushing failure

## Data Availability

The original contributions presented in this study are included in the article. Further inquiries can be directed to the corresponding authors.
